# Epstein-Barr-Virus-Driven Cardiolipin Synthesis Sustains Metabolic Remodeling During B-cell Lymphomagenesis

**DOI:** 10.21203/rs.3.rs-4013392/v1

**Published:** 2024-04-08

**Authors:** Haixi You, Larissa Havey, Zhixuan Li, John Asara, Rui Guo

**Affiliations:** Department of Molecular Biology and Microbiology, Tufts University, Boston, MA, USA; Department of Molecular Biology and Microbiology, Tufts University, Boston, MA, USA; Division of Infectious Diseases, Department of Medicine, Brigham and Women’s Hospital, 181 Longwood Avenue, Boston MA, USA; Division of Signal Transduction, Beth Israel Deaconess Medical Center and Department of Medicine, Harvard Medical School, Boston, MA, USA; Department of Molecular Biology and Microbiology, Tufts University, Boston, MA, USA

**Keywords:** B-cell transformation, cardiolipin synthesis, mitochondrial remodeling, aspartate, redox defense, metabolomics, isotope tracing, electron microscopy, tumor virus, virus oncoprotein, synthetic lethal strategies

## Abstract

Epstein-Barr Virus (EBV) is associated with a range of B-cell malignancies, including Burkitt, Hodgkin, post-transplant, and AIDS-related lymphomas. Studies highlight EBV’s transformative capability to induce oncometabolism in B-cells to support energy, biosynthetic precursors, and redox equivalents necessary for transition from quiescent to proliferation. Mitochondrial dysfunction presents an intrinsic barrier to EBV B-cell immortalization. Yet, how EBV maintains B-cell mitochondrial function and metabolic fluxes remains unclear. Here we show that EBV boosts cardiolipin(CL) biosynthesis, essential for mitochondrial cristae biogenesis, via EBNA2-induced CL enzyme transactivation. Pharmaceutical and CRISPR genetic analyses underscore the essentiality of CL biosynthesis in EBV-transformed B-cells. Metabolomic and isotopic tracing highlight CL’s role in sustaining respiration, one-carbon metabolism, and aspartate synthesis, all vital for EBV-transformed B-cells. Targeting CL biosynthesis destabilizes mitochondrial one-carbon enzymes, causing synthetic lethality when coupled with a SHMT1/2 inhibitor. We demonstrate EBV-induced CL metabolism as a therapeutic target, offering new strategies against EBV-associated B-cell malignancies.

## Introduction

Epstein-Barr Virus (EBV), infecting over 95% of the adult population worldwide, is a ubiquitous pathogen associated with a spectrum of diseases ranging from the benign infectious mononucleosis to more than 200,000 cases of cancer annually^1^. Among these, EBV-positive Burkitt lymphoma (BL) represents the most prevalent childhood cancer in Africa. Furthermore, EBV has been implicated in a variety of other malignancies, including Hodgkin lymphoma (HL), diffuse large B-cell lymphoma (DLBCL), nasopharyngeal carcinoma (NPC), and gastric carcinoma (GC)^2–7^. EBV immortalizes B-cells in vitro. The transformative ability plays a crucial role in the pathogenesis, especially in immunocompromised individuals such as organ transplant recipients and AIDS patients, where the incidence of EBV-associated lymphomas is notably high^8, 9^.

EBV actively modulates host metabolic pathways to support the survival and proliferation of transformed cells. This modulation is orchestrated through a complex interplay of viral-encoded oncogenes, including six EBV nuclear antigens (EBNA1, EBNA2, EBNA 3A-C, and EBNA-LP) and two latent membrane proteins (LMP1 and LMP2), along with various viral non-coding RNAs (ncRNAs). These viral factors initiate a cascade of metabolic reprogramming early in the infection process, leading to significant reorganization in B-cell architecture that favors cell growth and division. The early expression of EBNA2 and its primary target, MYC, in particular, activates multiple metabolic pathways including oxidative phosphorylation (Ox-Phos), one-carbon (1C) metabolism and sterol biosynthesis, further compounded by the expression of LMP1 and LMP2 which mimic activated CD40 and B-cell receptor pathways, respectively^10, 11^. The ability of EBV to reprogram host metabolism extends deeply into mitochondrial functions^12–20^, where it ensures the continuous generation of ATP and metabolic precursors critical for the synthesis of essential macromolecules and regulation of redox balance. Any perturbations in mitochondrial pathways, including Ox-Phos, the tricarboxylic acid (TCA) cycle, and the mitochondrial 1C metabolism, is detrimental to the EBV transformation process^12, 17, 21^. Metabolic stress significantly hinders the transformation of B cells by EBV; newly infected cells that do not undergo successful transformation are characterized by mitochondrial dysfunction^17^. However, the mechanisms through which EBV sustains mitochondrial function and metabolic fluxes during these early transformation stages remain elusive.

Cardiolipin (CL) is a four-acyl chain lipid that exists exclusively in the inner mitochondrial membrane (IMM), which plays an important role in the mitochondrial function and constitutes about 20% of the total mass of phospholipids of IMM. CL is essential for shaping the mitochondrial cristae and binding to electron transport chain (ETC) complexes in the IMM^22, 23^. Defects in CL metabolism led to compromised respiration and disruptions in the TCA cycle^22–24^. CL also modulates apoptosis by controlling cytochrome c release^25^. Here we show how early EBV infection in B-cells exploits CL biosynthesis pathways to establish specialized mitochondria that sustain extensive metabolic network remodeling. Our findings highlight a key metabolic vulnerability in EBV’s transformation process and provide a foundational basis for developing synthetic lethal strategies to treat EBV-induced B-cell lymphoproliferative disorders.

## Results

### EBV upregulates CL biosynthesis and remodels B-cell mitochondrial ultrastructure

**Figure 1:**
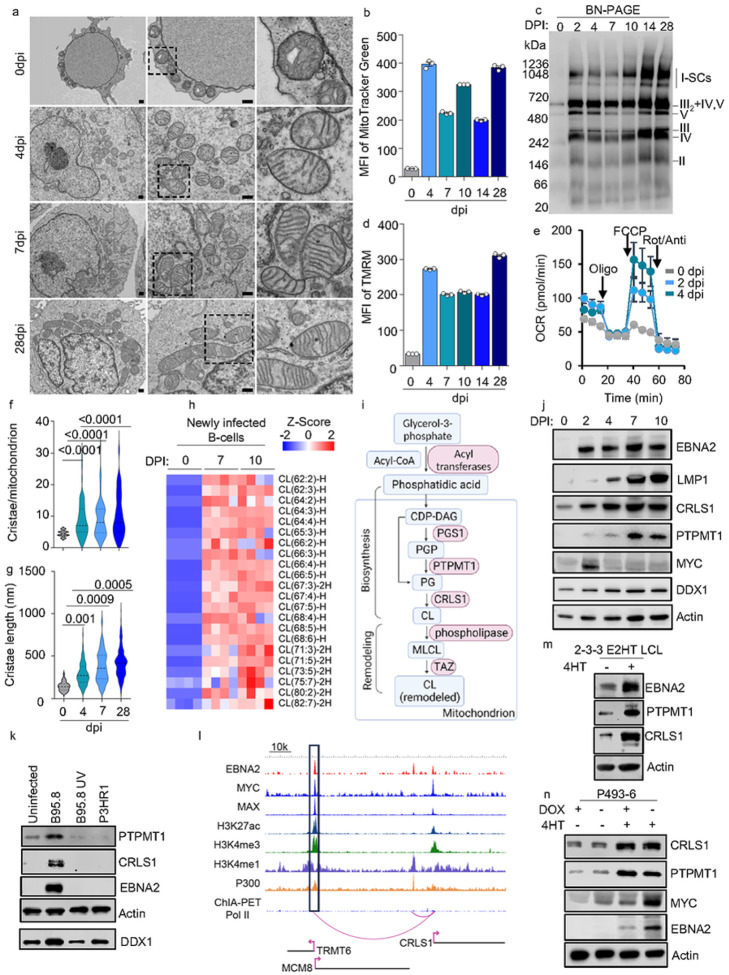
EBV regulates CL biosynthesis to sustain mitochondrial remodeling during B-cell transformation. a. TEM analysis of EBV newly infected B cells collected at 0-, 4-, 7-, and 28-days post-infection. Scale bar, 500nm. b. FACS analysis of Mitotracker Green mean fluorescence intensity (MFI) in newly transformed human primary B-cells by EBV, collected at the indicated days post-infection. Mean +/− SD values were from n=3 experiments. c. Immunoblot analysis of mitochondrial ETC complexes in EBV newly transformed human primary B-cells, collected at indicated days post-infection. BN-PAGE was used to separate the complexes from 20μg of freshly isolated mitochondria. Anti-human Ox-Phos antibody cocktail was used to visualize the ETC complexes. d. FACS analysis of TMRM mean fluorescence intensity (MFI) in EBV newly transformed human primary B-cells, collected at indicated days post-infection. Mean +/− SD values were from n=3 experiments. e. Mitochondrial stress test of uninfected or EBV newly infected B-cells, as indicated using a Seahorse analyzer. Mean +/−SD values were from n=3 technical repeats. The data shown was representative of n=3 experiments using different donors. f. Statistical analysis of cristae number per mitochondrial from 5 random TEM pictures as shown in 1a. P values were calculated using unpaired Student t-test. g. Statistical analysis of cristae length from 5 random TEM pictures as shown in 1a. P values were calculated using one-way ANOVA unpaired Student t-test. h. Heatmap visualization of z-scores of CL species detected in lipidomics analysis of uninfected or newly infected primary B-cells by EBV, collected at day 7 or day 10 post-infection. The total lipids were extracted from 5 million cells of each sample and quantified by LC/MS. The abundance of individual lipid species was normalized against the total ion count of the sample. The z-score describes the standard deviation variation from the mean value of each lipid. Individual data from n=4 samples are shown. i. Schematic picture of CL biosynthesis and remodeling. PGP, phosphatidyl glycerophosphate; PG, phosphatidylglycerol; CL, cardiolipin; CDP-DAG, CDP-diacylglycerol; MLCL, Monolysocardiolipin. Key enzymes are shown in red. j. Immunoblot analysis for indicated proteins in whole cell lysates (WCL) in EBV newly transformed human primary B-cells, collected at indicated days post-infection. k. Immunoblot analysis of WCL in uninfected, or B-cell infected with B95.8, UV-inactivated B95.8, or P3HR-1 viruses, collected at day 2 post-infection. Input viral genome was normalized by absolute quantification using qRT-PCR. l. ChIP-seq tracks of the indicated transcription factors, along with the activating histone epigenetic marks H3K27Ac, H3K4Me1, or H3K4Me3, at the GM12878 LCL CRLS1 locus are shown. Also displayed are the long-range DNA linkages between an upstream MCM8 locus enhancer and CRLS1, as defined by GM12878 ChIA-PET. m. Immunoblot analysis of EBNA2, PTPMT1, CRLS1, and actin expression in EBNA2-HT cells cultured in the presence or absence of 4HT (1 μM). n. Immunoblot analysis of EBNA2, PTPMT1, CRLS1, MYC and actin expression in P493–6 cells cultured with the indicated supplement. Blots in c, j, k, m, and n were representative of at least n=3 experiments.

A central hub for metabolic regulation in cells is the mitochondrion. Yet, how EBV regulates mitochondrial remodeling is not fully understood. To address this, we first investigated how EBV infection alters the mitochondrial ultrastructure to support B-cell transformation. To capture dynamic change of the mitochondrial ultrastructure upon EBV infection, a transmission electron microscopy (TEM) was used to analyze human B-cells, either uninfected or infected with EBV and collected at days 4, 7, and 28 post-infection (DPI). The infected cells appeared to be much larger in size with substantially increased cytosolic space (Fig. 1a). After EBV infection, there was a drastic increase in the number of mitochondria per cell, which was confirmed by a significant increase in the MitoTracker green signal and mtDNA abundance (Fig. 1a–b, **Extended Data** Fig. 1a). Analyzing a published RNAseq dataset of B-cells infected with EBV reveals a significant increase in the transcription of genes involved in the ETC^26^. 2DPI cells exhibited the highest transcriptional changes in these ETC genes (**Extended Data** Fig. 1c). Gene ontology analysis of differentially expressed metabolic genes at 2DPI reveals terms including cellular respiration and respiratory chain complex assembly (**Extended Data** Fig. 1c). We therefore employed blue native polyacrylamide gel electrophoresis (BN-PAGE) to visualize mitochondrial respirasome assembly during EBV infection in B-cells. Compared to uninfected human resting B-cells, ETC supercomplexes in EBV-infected cells were readily detected from 20 μg of mitochondrial proteins starting from 2 DPI and onwards (Fig. 1c). From 10DPI, an additional increase of complex I and III_2_ + IV + V supercomplexes was observed (Fig. 1c). Consistently, increases in mitochondrial membrane potential, basal and maximal oxygen consumption rates, as well as ATP production, were significantly elevated from 2 DPI (Fig. 1d–e, **Extended Data** Fig. 1d). These findings suggest that EBV strongly regulates mitochondrial biogenesis, ETC supercomplex assembly, and respiration at the early stage of infection.

Along with the substantial increase in mitochondrial numbers following EBV infection, we observed a large boost in mitochondrial cristae biogenesis, as indicated by both the increased number per mitochondrion and extended length of cristae (Fig. 1a, 1f–g). Cristae are distinguished by their membrane invagination structures that project into the mitochondrial matrix. These IMM folds greatly expand the mitochondrial inner membrane’s surface area^27^. CL plays a crucial role in shaping the curvature of cristae and exhibits a strong affinity for numerous mitochondrial supercomplexes, including the respirasome^24^. Our untargeted lipidomic analysis using Liquid chromatography–mass spectrometry (LC/MS) revealed a remarkable elevation in various CL species in EBV-infected B-cells at 7 or 10 DPI compared to uninfected resting B-cells (Fig. 1h). Based on these observations, we hypothesize that the upregulation of CL biosynthesis acts as a contributing factor in the mitochondrial remodeling induced by EBV, thereby facilitating key metabolic pathways driven by EBV.

The CL biosynthetic pathway involves a cascade of enzymatic reactions, including key enzymes such as protein tyrosine phosphatase mitochondrial 1 (PTPMT1) and cardiolipin synthase 1 (CRLS1)^22^. These enzymes work sequentially to convert phosphatidic acid into nascent CL (Fig. 1i). EBV robustly upregulated CRLS1 transcription and translation by 2 DPI, coinciding with the heightened expression of EBNA2 and MYC (Fig. 1j, **Extended Data** Fig. 1e). PTPMT1 protein was slightly increased at 2 DPI, and largely enhanced from 7 DPI, coinciding with the increase in LMP1 levels (Fig. 1j). By contrast, the PTPMT1 mRNA was modestly increased by 2 DPI and remained at a similar level as uninfected cells after 7DPI suggesting the post-transcriptional regulation of its expression, potentially regulated by LMP1^26^ (**Extended Data** Fig. 1e).

Recognizing EBNA2 as a key viral regulator in B cell metabolism^12, 28^, our study focused on its impact in initiating CL biosynthesis. We infected primary human B cells with either the B95.8 strain, or UV-inactivated B95.8 or the non-transforming P3HR-1 strain of EBV^29, 30^ (Fig. 1k). To maintain consistent infection levels across samples, we adjusted for input viral genome copy numbers, basing these adjustments on quantitative PCR analyses of the virus stocks (**Extended Data** Fig. 1f). Notably, the P3HR-1 strain lacks EBNA2 and most EBNA-LP open reading frames making it an ideal tool virus to study EBNA2 and/or EBNA-LP function^31^. The immunoblot revealed that only the B95.8 strain, but neither the P3HR-1 nor UV-inactivated B95.8, was capable of triggering the expression of CRLS1 and PTPMT1 by 2DPI. This observation was made while ensuring equal levels of infection, as evidenced by comparable post-infection intracellular viral loads (**Extended Data** Fig. 1g). These findings indicate that EBV EBNA2 and/or EBNA-LP, rather than a broad response to EBV infection, are crucial for triggering cardiolipin biosynthesis.

In an effort to further understand the potential roles of EBNA2 in the activation of CRLS1, the rate limiting enzyme in CL biosynthesis, we utilized public available resources including ENCODE GM12878 LCL ChIP-seq dataset^32^, EBV EBNA2 ChIP-seq dataset^33^ and long-range chromatin interaction analysis^34^. We discovered that EBNA2, along with its host targets MYC, co-occupy the promoter of CRLS1 (Fig. 1l). The 2-2-3 LCL cell line expresses an engineered EBNA2, where EBNA2 is fused to a modified estrogen receptor ligand-binding domain. Therefore, EBNA2 transcriptional activity can be regulated by the presence of 4-hydroxytamoxifen (4HT). With 4HT, EBNA2–4HT is stabilized and translocated to the nucleus and regulates transcription^35^. When EBNA2 activity is conditionally shut down by withdrawing 4HT for a period of 48 hours, there is a noticeable reduction in the levels of CRLS1 and PTPMT1 (Fig. 1m).

It’s well-established that EBNA2 and MYC collaboratively stimulate EBV target metabolic genes necessary for B-cell transformation^33, 34^. ChIP-seq in LCLs showed that MYC and MAX, which form a heterodimer complex binding to E-box sites, co-occupied the LCL CRLS1 promoter (Fig. 1l). Next, we investigated MYC’s role in EBV-induced CL metabolism. Using the P493–6 B cell line, an LCL with a 4HT-inducible EBNA2 cassette and a tetracycline-regulated MYC cassette, we found that merely activating MYC by withdrawing doxycycline could not induce CRLS1 expression, whereas activation of EBNA2 by adding 4HT was sufficient to induce CRLS1 expression (Fig. 1m). This suggests that CRLS1 is an EBNA2-specific target.

We also found EBNA2 and MYC in an upstream enhancer (about 110kbp away), marked by H3K27ac, H3K4me1, and EP300 co-ocupancy, linked to the CRLS1 promoter by a chromatin long range loop (Fig. 1l, **rectangle**). Using CRISPR interference (CRISPRi) on GM12878 LCL cells, we were able to disrupt the CRLS1 enhancer. GM12878 LCL expressing dCas9-repressor and CRLS1 enhancer targeted sgRNAs showed significantly reduced CRLS1 expression at both mRNA and protein level (**Extended Data** Fig. 1h–i). Overall, our data reveals an EBV-specific transcriptional regulation mechanism for rate-limiting CL metabolic enzymes orchestrated by viral oncoprotein EBNA2.

### CL biosynthesis is critical for EBV B-cell transformation and LCL survival.

**Figure 2:**
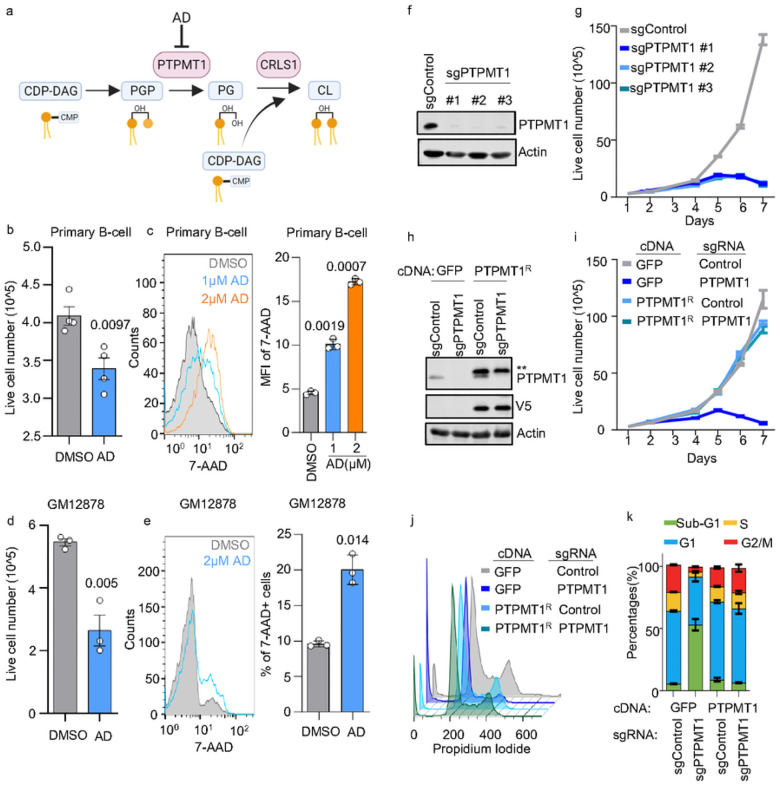
Inhibiting CL biosynthesis impedes EBV-transformed B-cells growth. a. Schematic showing that alexidine dihydrochloride (AD) selectively inhibits PTPMT1, which catalyzes the conversion of PGP to PG, a precursor of CL. b. Live cell number of EBV infected 4DPI primary B-cells that treated with DMSO control or 2μM of AD for 24 hours. Cells were cultured at an indensity of 3×10^5/mL cells. Mean +/− SD values were from n=4 experiments using different donors. P-values were calculated using unpaired Student t-test. c. FACS analysis of 7-AAD in EBV infected 4DPI primary B-cells that treated with DMSO control, 1 or 2μM of AD for 24hours. 7-AAD MFI were shown using mean +/− SD values from n=3 experiments. P-values were calculated using a paired Student t-test. d. Live cell number of GM12878 LCLs that treated with DMSO control or 2μM of AD for 24 hours. Cells were cultured at an indensity of 3×10^5/mL cells. Mean +/− SD values were from n=3 experiments. P-values were calculated using unpaired Student t test. e. FACS analysis of 7-AAD in GM12878 LCLs that treated with DMSO, or 2μM of AD for 24 hours. 7-AAD MFI were shown using mean +/− SD values from n=3 experiments. P-values were calculated using a paired Student t-test. f. Immunoblot analysis of PTPMT1 or Actin in WCL of Cas9+ GM12878 LCL expressing control or indicated PTPMT1 sgRNAs. g. Growth curves of Cas9+ GM12878 LCL expressing control or indicated PTPMT1 sgRNAs. Mean +/− SD values were from n=3 experiments. h. Immunoblot analysis of WCL from Cas9+ GM12878 LCL following expression of the indicated GFP control or PTPMT1R rescue cDNAs and the indicated control or PTPMT2-targeting sgRNAs. Representative blot of n = 3 replicates shown. i. Growth curves of Cas9+ GM12878 LCL following expression of the indicated GFP control or PTPMT1R rescue cDNAs and the indicated control or PTPMT2-targeting sgRNAs. Mean +/− SD values were from n=3 experiments. j. Cell cycle analysis of Cas9+ GM12878 LCL following expression of the indicated GFP control or PTPMT1R rescue cDNAs and the indicated control or PTPMT2-targeting sgRNAs. k. Percentage of Sub-G1, G1, S, and G2/M phase in cell cycle analysis in j. Mean +/− SD values were from n=3 experiments. Blots in f and h were representative of at least n=3 experiments.

PTPMT1 converts phosphatidylglycerophosphate (PGP) to phosphatidylglycerol (PG). Subsequently, CRLS1 finalizes the process by transforming phosphatidylglycerol (PG) and cytidine diphosphate-diacylglycerol (CDP-DAG) into cardiolipin^36^ (Fig. 2a). Alexidine dihydrochloride (AD) is a highly selective PTPMT1 inhibitor^37^. AD treatment on EBV newly infected B-cells significantly hampered transformation and increased 7-Aminoactinomycin D (7-AAD) cell death signal on a dose dependent manner (Fig. 2b–c). Similar AD induced growth defects were observed in GM12878 and other four LCLs (Fig. 2de, **Extended Data** Fig. 2a). To further confirm the AD effects, we employed CRISPR/Cas9 system to knock-out the PTPMT1 in several LCLs. We achieved > 95% of PTPMT1 knockout efficiency with all three individual sgRNA (Fig. 2f), which consistently diminished GM12878 growth (Fig. 2g). Similar growth defects were observed in GM12881 Cas9 + and GM12892 Cas9 + LCLs expressing PTPMT1 sgRNAs (**Extended Data Fig. S2b-c**). Importantly, when replacing a PAM-site disabled PTPMT1 cDNA in PTPMT1 KO GM12878 cells (Fig. 2h), proliferation deficiency led by endogenous PTPMT1 loss was fully rescued (Fig. 2i). Propidium iodide cell cycle analysis demonstrated that PTPMT1 significantly increased the sub-G1 population and decreased the G1, S, and G2/M population, indicative of cell death, while the PAM-site mutated PTPMT1 can rescue the KO effects (Fig. 2j–k). We further tested the effects of PTPMT1 KO in BLs, a major EBV-associated, AIDS-related lymphoma. Similar growth defects were observed in PTPMT1 KO P3HR-1 and Rael BLs (**Extended Data Fig. S2d–e**). Collectively, these data suggest that the survival of EBV-transformed B-cells and BLs depends on a functional CL biosynthetic pathway.

### Impaired CL biosynthesis disrupts mitochondrial respiration in EBV-transformed B-cells.

**Figure 3:**
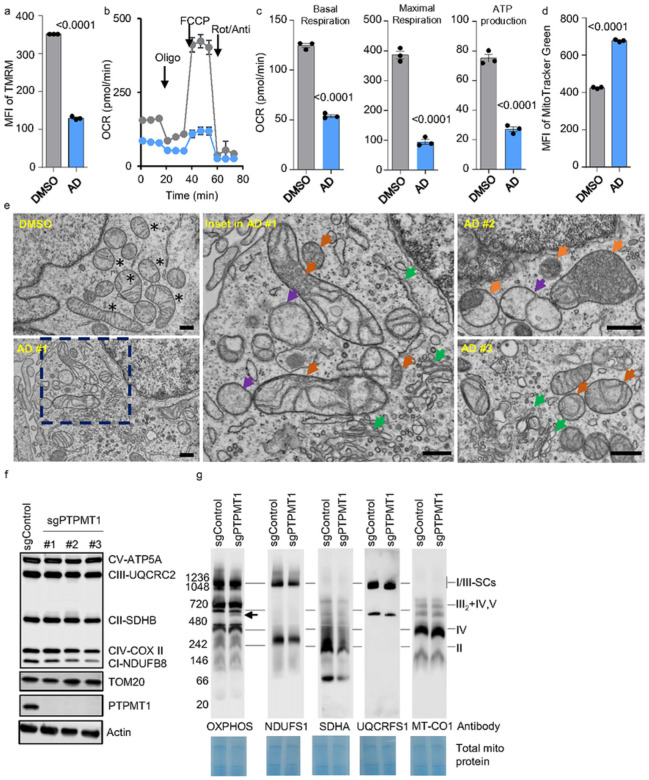
CL inhibition disrupts respiration, cristae biogenesis, and complex II assembly. a. FACS analysis of TMRM MFI in EBV infected 4DPI primary B-cells treated with DMSO or 2μM of AD for 24 hours. 4DPI primary B-cells cells were seeded at 3×10^5/mL. Mean +/− SD values were from n=3 experiments. P-values were calculated using an unpaired Student’s t-test. b. Mitochondrial stress test of EBV infected 4DPI primary B-cells treated with DMSO or 2μM of AD for 24 hours using a Seahorse analyzer. 4DPI primary B-cells cells were seeded at 3×10^5/mL. Mean +/− SD values were from n=3 experiments. c. Seahorse oxygen consumption rate (OCR) linked to basal respiration, maximal respiration, and ATP production in uninfected or infected primary B-cells collected at 4DPI. Mean +/− SD values were from n=3 experiments. P-values were calculated using an unpaired Student’s t-test. d. FACS analysis of MitoTracker Green MFI in EBV infected 4DPI primary B-cells treated with DMSO or 2μM of AD for 24 hours. 4DPI primary B-cells cells were seeded at 3×10^5/mL. Mean +/− SD values were from n=3 experiments. P-values were calculated using an unpaired Student’s t-test. e. TEM analysis of EBV infected 4DPI primary B-cells treated with DMSO or 2μM of AD for 24 hours. Scale bar, 500nm. Top left panel (DMSO), a representative field of DMSO-treated cells. Bottom left (AD#1) and two right panels (AD#2 and #3), representative of AD-treated cells. Middle panel, the inset field in bottom left panel. Black asterisks, healthy mitochondria with organized cristae; orange arrowheads, disordered cristae accumulating in mitochondria; purple arrowheads, empty mitochondria; green arrowheads, increased ER membrane and Golgi apparatus. Scale bar, 500nm. f. SDS-PAGE and Immunoblot analysis using Ox-Phos antibody cocktail, TOM20, PTPMT1, CRLS1, or Actin antibody in WCL from Cas9+ GM12878 LCL following expression of the indicated GFP control or PTPMT1R rescue cDNAs and the indicated control or PTPMT2-targeting sgRNAs. Representative blot of n = 3 replicates shown. g. BN-PAGE and Immunoblot analysis using Ox-Phos antibody cocktail, NDUFS1 (complex I), SDHA (complex II), UQCRCS1 (complex III), or MT-CO1 (complex IV) antibody of 20μg of freshly isolated mitochondria from Cas9+ GM12878 LCL expressing control or PTPMT1 sgRNA. Total input protein was measured by Coomassie blue G-250 staining of the transferred membrane for immunoblot.

EBV-driven B-cell transformation relies on functional Ox-Phos^12, 28^. Selectively inhibiting the electron transport chain halts EBV-driven B-cell transformation^12^. Given the essential role of CL in maintaining mitochondrial function, we first investigated whether disrupting CL biosynthesis impacts mitochondrial respiration in cells newly infected with EBV. As expected, treatment with AD significantly reduced the B-cell mitochondrial membrane potential, as measured by TMRM, at 4 DPI (Fig. 3a, **Extended Data** Fig. 3a–b). Along with this, the oxygen consumption rates (OCRs) of both basal and maximal respiration, as well as ATP production, were significantly reduced (Fig. 3b–c). Notably, this inhibition of Ox-Phos is not attributable to decreased mitochondrial biogenesis; the level of MitoTracker Green was significantly increased after AD treatment (Fig. 3d, **Extended Data** Fig. 3c–d).

Given CL contributes to mitochondrial cristae, we hypothesize that AD treatment may disrupt cristae structure in EBV newly infected B-cells. We therefore performed TEM analysis on 4DPI cells that were treated with DMSO or AD for 24h (Fig. 3e, **Extended Data** Fig. 3e). In DMSO treated cells, the cristae are observed as shelf-like invaginations extending into the mitochondrial matrix (Fig. 3e, **Extended Data** Fig. 3e). They generally display a lamellar shape but sometimes appear to be short sphere which is probably due to ongoing cristae remodeling (asterisks). By contrast, in AD treated cells (Fig. 3e), the number of “empty” mitochondria lacking cristae was greatly increased (purple arrows). In some mitochondria, we observed a highly disorganized cristae structure which is usually located at one side of the mitochondria (orange arrows) and multiple long and distorted tube-like structures originate from it (orange arrows). Besides the defects in mitochondria, we also observed an increased number of intracellular vesicles, endoplasmic reticulum, and Golgi apparatus in AD treated cells (green arrows).

CL stabilizes the electron transport chain (ETC) supercomplexes located on the cristae^22, 27, 36, 38^. We then investigated whether PTPMT1 deficiency destabilizes the respiration supercomplexes in EBV-transformed LCLs. We knocked out PTPMT1 in GM12878 cells and subsequently tested the abundance of ETC proteins from whole cell lysates using an Ox-Phos antibody cocktail. Overall, SDHB in complex II, UQCRC2 in complex III, COX II in complex IV, and ATP5A in complex V remained stable in PTPMT1 KO GM12878 LCLs. Interestingly, we observed a noticeable downregulation of NDUFB8 in complex I in PTPMT1 KO cells expressing two of the PTPMT1 sgRNAs(Fig. 3f). We further investigated whether PTPMT1 deficiency might impact the assembly of ETC supercomplexes. Therefore, we isolated mitochondria from control and PTPMT1 KO GM12878 LCLs and performed BN-PAGE and then immunoblotting for ETC complexes. In this experiment, immunoblotting with the anti-Ox-Phos antibody cocktail revealed an additional unknown band with a size of around 550 kDa, as indicated by an arrow (Fig. 3g). Other than this, we did not observe any obvious differences between the control and PTPMT1 KO cells using anti-Ox-Phos antibody cocktail. However, when using individual antibodies targeting individual ETC complex, we found a striking reduction of SDHA in complex II, suggesting that PTPMT1 deficiency may disrupt SDHA assembly in complex II in EBV LCLs(Fig. 3g).

### Impaired CL biosynthesis leads to NADPH deficiency and oxidative stress in EBV-transformed B-cells.

**Figure 4:**
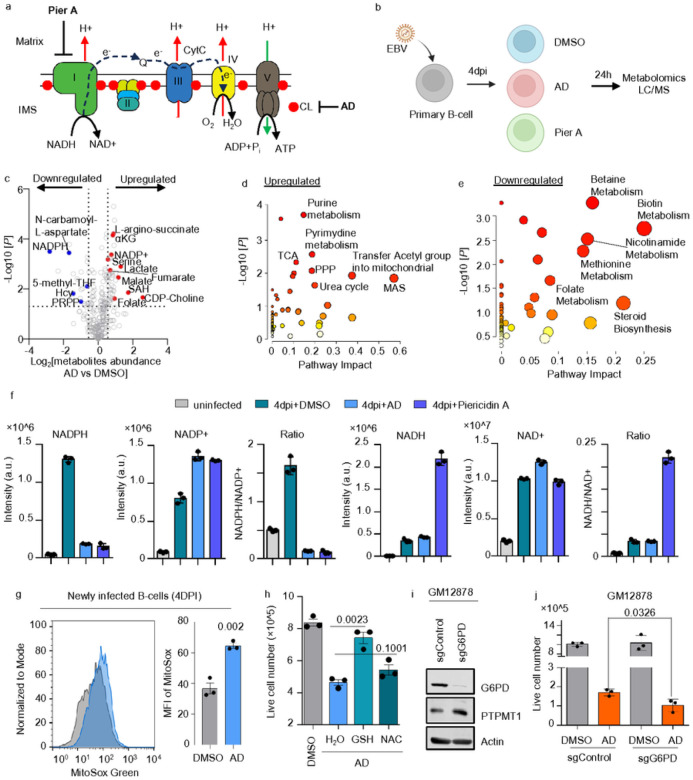
CL inhibition disrupts intermediary metabolism and NADPH production in EBV newly infected B-cells. a. Schematic representation of ETC complexes stabilized by cardiolipin (CL). Piericidin A (PierA) acts as a selective inhibitor of Complex I (NADH dehydrogenase). Alexidine dihydrochloride (AD) selectively inhibits PTPMT1, affecting CL biosynthesis. b. Human primary B cells, freshly isolated, were infected with EBV at a MOI of 1. Four days post-infection, treatments with DMSO, 2μM AD, or 0.1μM PierA were applied for an additional 24 hours. LC/MS was used for untargeted metabolomics to quantify intracellular metabolites. c. Volcano plot comparing metabolomic profiles of primary B cells, 4 days post-infection (DPI), treated with 1μM compound versus DMSO for 24 hours, based on data from three replicates. P-values determined by Student’s t-test, assuming unequal variances between samples. d. Metabolic pathway analysis highlighting pathways significantly upregulated by AD treatment. The x-axis shows pathway impact values from MetaboAnalyst 3.0 topological analysis; the y-axis shows −log of P-value from pathway enrichment analysis. e. Metabolic pathway analysis highlighting pathways significantly downregulated by AD treatment. Axes as described in (d). f. Bar chart showing levels of NADPH, NADP+, NADH, and NAD+, including ratios of NADPH/NADP+ and NADH/NAD+, demonstrating changes in cellular redox states. g. FACS analysis of MitoSox Green fluorescence in primary B cells, 4 DPI, treated with DMSO or 1μM AD for 36 hours. Data represents mean ± SD from three experiments. P-values calculated using unpaired Student’s t-test. h. Absolute number of live EBV-infected primary B cells, 4 DPI, treated with DMSO or 2μM AD, supplemented with H2O, an unspecified amount of 2 mM GSH, or 1.5 mMNAC for 24 h. Data represent mean ± SD from three experiments, with P-values from unpaired Student’s t-test. i. Immunoblot analysis for G6PD, PTPMT1, and Actin in WCL of Cas9+ GM15892 LCL expressing control or G6PD sgRNAs. Results are representative of three experiments. j. Absolute number of live cells in control or G6PD knockout (KO) Cas9+ GM12878 LCL treated with DMSO or 2 μM AD for 48 h. Data represent mean ± SD from three experiments, with P-values from unpaired Student’s t-test.

To elucidate the metabolic impacts of AD treatment on newly EBV-infected B-cells, we further employed an LC/MS based untargeted metabolomic analysis. We used Piericidin A (PierA) as a comparative control, which is known for its selective inhibition of complex I, thereby hindering mitochondrial respiration (Fig. 4a). Freshly isolated human primary B cells were infected with EBV B95.8 at a multiplicity of infection (MOI) of 1. At 4DPI, the cells were treated with either DMSO, AD, or PierA for an additional 24 hours. Subsequent LC/MS-based metabolomic analyses of these samples unveiled significant variances in intracellular metabolites across cells treated with DMSO, AD, or PierA (**Extended Data** Figs. 4 and 5a). Relative to DMSO-treated cells, AD treatment led to an upregulation of metabolites within the B-cell malate aspartate shuttle, the TCA cycle, the pentose phosphate pathway (PPP) and purine and pyrimidine metabolism. In contrast, it downregulated metabolites associated with sterol synthesis, as well as folate, methionine, betaine, and nicotinamide metabolism (Fig. 4c–e). PierA treatment, on the other hand, significantly downregulated metabolites in pyrimidine metabolism and the TCA cycle, while upregulating those in purine metabolism and contributing to the Warburg effect (**Extended Data** Fig. 5b–c). Despite some overall correlation in the metabolic profiles between AD and PierA-treated cells, we identified notable exceptions: levels of citrate, succinate, itaconate, orotate, and glucosamine were increased in AD-treated cells but were significantly decreased in cells treated with PierA (**Extended Data** Fig. 5b, 5d).

Notably, AD treatment significantly reduced NADPH levels by 7-fold and increased NADP + by 1.5-fold, leading to a marked decrease in the NADPH/NAD + ratio (Fig. 4f). This change disrupts cellular redox balance and impairs biosynthetic pathways, including sterol synthesis and folate metabolism (Fig. 4c). We observed increased mitochondrial ROS in AD-treated B-cells, indicating elevated oxidative stress (Fig. 4g). Glutathione (GSH) supplementation reduced AD-induced cell mortality effectively, whereas N-acetyl-cysteine (NAC) was less effective (Fig. 4h). Unlike PierA treatment, AD did not affect NADH and NAD + levels, suggesting a targeted disruption of anabolic processes with little impact on catabolic processes like TCA cycle. This specificity indicates CL’s role in modulating anabolic and oxidative stress responses.

The PPP is the major cytosolic pathway generating NADPH. We further tested if PPP plays a potential role in replenishing NADPH in AD-treated cells. We therefore knocked out Glucose-6-phosphate dehydrogenase (G6PD), the rate-limiting enzyme in the PPP. Interestingly, our immunoblotting revealed an unexpected negative correlation between G6PD and PTPMT1 in GM12878 LCL (Fig. 4i), suggesting potential feedback between CL biosynthesis and PPP. We found G6PD KO itself did not affect cell growth in GM12878 LCL. When combined with AD treatment, G6PD KO led to an evident synthetic lethality (Fig. 4j). Collectively, these results suggest that inhibiting CL decreases NADPH levels and leads to cell death dependent on oxidative stress, which can be mitigated by the exogenous supplementation of GSH.

### Impaired CL biosynthesis disrupts mitochondrial 1C metabolism in EBV-transformed B-cells

**Figure 5:**
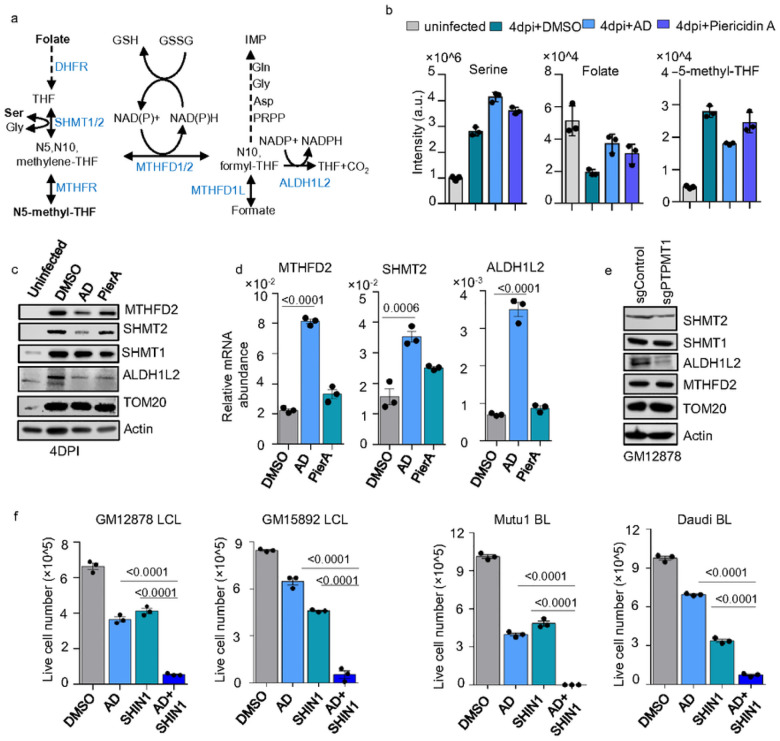
Inhibiting CL biosynthesis disrupts 1C metabolism by destabilizing mitochondrial 1C enzymes in EBV transformed B-cells. a. Schematic of 1C metabolism showing it NADPH production and supports IMP synthesis. Enzymes are shown in blue. b. Bar chart showing levels of serine, folate, and 5-methyl-THF. Data represent mean ± SD from three experiments. c. Immunoblot analysis for MTHFD2, SHMT2, ALDH1L2, SHMT1, TOM20 and Actin in WCL of uninfected, or EBV-infected primary B cells at 4DPI, treated with DMSO, 2μM of AD, or 0.1 μM of PierA for 24 hours. Results are representative of three experiments. d. Quantitative RT-PCR for MTHFD2, SHMT2, or ALDH1A2 mRNA abundance in EBV-infected primary B cells at 4DPI, treated with DMSO, AD, or PierA as indicated in c. Data represent mean ± SD, P values were calculated using one-way ANOVA with Tukey’s multiple comparisons test. e. Immunoblot analysis for MTHFD2, SHMT2, ALDH1L2, SHMT1, TOM20 and Actin in WCL of GM12878 LCL expressing control or PTPMT1 sgRNAs. Results are representative of three experiments. f. Absolute live number of GM12878 LCL, GM15892 LCL, Mutu I BL, or Daudi BL, treated with DMSO, 1μM AD, 10μM SHIN1, or 1μM AD+10μM SHIN1 for 48h. Data represent mean ± SD from three experiments, with P-values from unpaired Student’s t-test.

1C metabolism catalyzes the transfer of 1C units across various metabolites. A significant part of this process occurs in mitochondria, where key enzymes like SHMT2 and MTHFD2 catalyze reactions using folate as a one-carbon carrier^12^. EBV B-cell transformation activates and heavily relies on this metabolic pathway, which primarily contributes to NADPH, as well as purine and thymidylate synthesis (Fig. 5a)^12, 39^. We found AD treatment in newly infected B-cells leads to an accumulation of intracellular serine and folate, while downstream product 5-methyl-THF is significantly reduced (Fig. 5b, **Extended Data** Fig. 4) suggesting the disruption in 1C metabolism. Interestingly, in cells treated with AD, there was a significant reduction in the protein levels of SHMT2 and MTHFD2, key enzymes in the mitochondrial 1C pathway (Fig. 5c). The conversion of 10-formyl-THF to THF and CO_2_ by ALDH1L2 contributes to the pool of NADPH (Fig. 5a). Interestingly, ALDH1L2 protein was reduced in both AD- and PierA-treated cells (Fig. 5c). By contrast, the cytosolic SHMT1 and mitochondrial outer membrane protein TOMM20 were not affected by AD treatment (Fig. 5c). Quantitative RT-PCR results showed that AD treatment, in fact, slightly increased the transcription of MTHFD2, SHMT2, and ALDH1L2 (Fig. 5d). Additionally, the destabilization of SHMT2 and ALDH1L2 proteins was observed in PTPMT1 knockout GM12878 LCLs, whereas MTHFD2 levels remained unchanged (Fig. 5e). These data suggest that CL inhibition may destabilize the mitochondrial 1C enzymes. Therefore, we posited that targeting CL biosynthesis could potentially synergize with pharmaceutical inhibition of 1C metabolism to achieve synthetic lethality. SHIN1, a highly selective small molecular inhibitor that simultaneously blocks SHMT1 and SHMT2^40^, has been shown to impair EBV B-cell transformation^12^. Importantly, we found that SHIN1 exhibits a striking synthetic lethal effect when combined with AD treatment in GM12878 and GM15892 LCLs. Similar effects were observed in Mutu I and Daudi BLs (Fig. 5f).

### Impaired CL biosynthesis leads to dysfunction of GOT2-driven aspartate biosynthesis in EBV-transformed B-cells.

**Figure 6:**
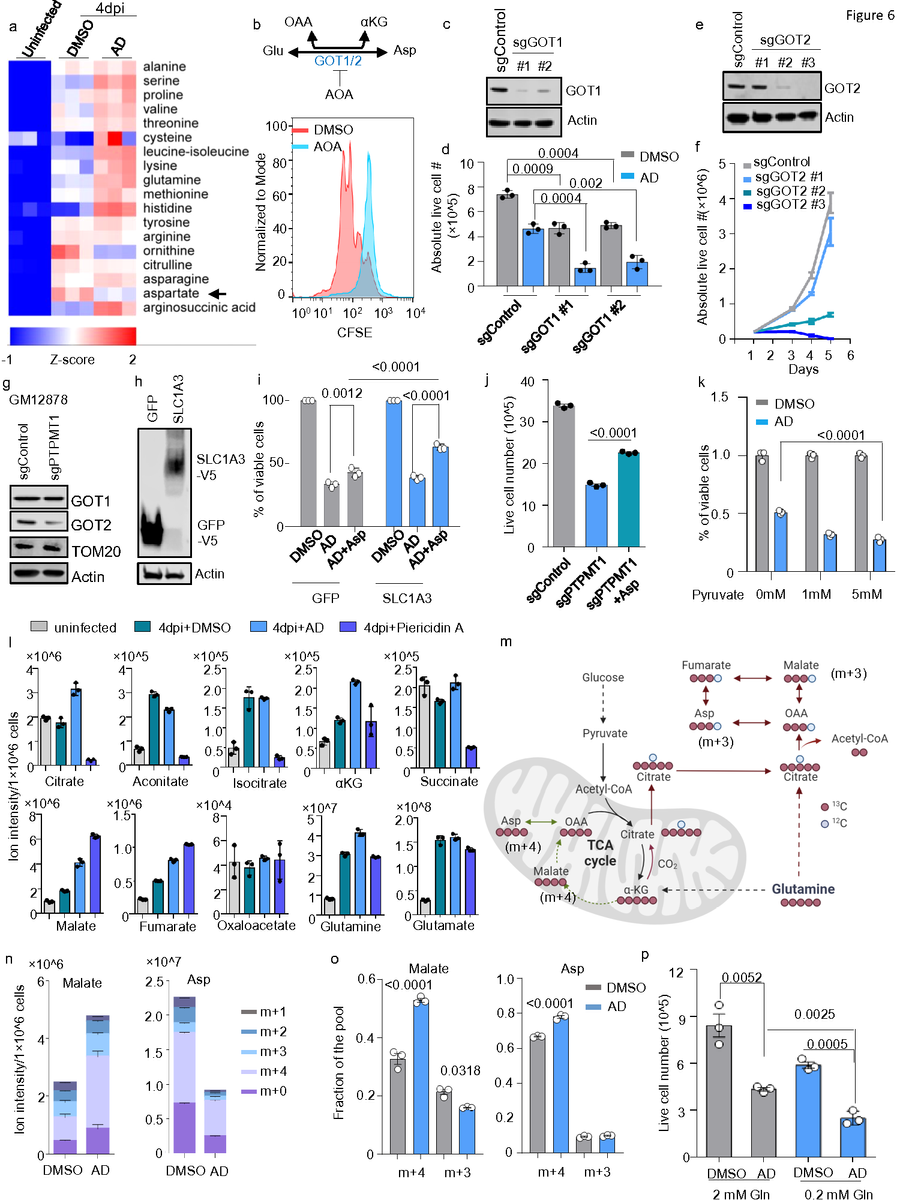
Inhibiting CL biosynthesis disrupts aspartate synthesis in EBV transformed B-cells. a. Heatmap visualization of z-scores of amino acids detected in metabolomic analysis of uninfected, or EBV-infected primary B cells at 4DPI, treated with DMSO, 2μM of AD. The z-score describes the standard deviation variation from the mean value of each gene. Individual data from n=3 samples are shown. b. FACS CSFE dye-dilution analysis of EBV newly infected primary human B cells, treated with DMSO, or 100μM AOA, a GOT1/2 dual inhibitor for 5 days. Representative of n=3 replicates. c. Immunoblot analysis for GOT1 and Actin in WCL of Cas9+ GM12878 LCL expressing control or indicated GOT1 sgRNAs. Results are representative of three experiments. d. Absolute live cell number of control or GOT1 knockout (KO) Cas9+ GM12878 LCL treated with DMSO or 1μM AD for 48h. Data represent mean ± SD from three experiments, with P-values from unpaired Student’s t-test. e. Immunoblot analysis for GOT2 and Actin in WCL of Cas9+ GM12878 LCL expressing control or indicated GOT2 sgRNAs. Results are representative of three experiments. f. Growth curves of Cas9+ GM12878 LCL expressing control or indicated GOT2 sgRNAs. Mean +/− SD values were from n=3 experiments. g. Immunoblot analysis for GOT1, GOT2, TOM20 and Actin in WCL of GM12878 LCL expressing control or PTPMT1 sgRNAs. Results are representative of three experiments. h. Immunoblot analysis for V5 tagged protein and Actin in WCL of Cas9+ GM12878 LCL expressing GFP or SLC1A3. Results are representative of three experiments. i. Percentage of live cell number of GM12878 LCL expressing GFP or SLC1A3, treated with DMSO or 1μM AD, or 1μM AD+1mM Asp for 48h. Data represent mean ± SD from three experiments, with P-values from 2way ANOVA by Tukey’s multiple comparisons test. j. Live cell number of Cas9+ GM12878 LCL expressing control or PTPMT1 sgRNA cultured with or without Asp. At day 3 post-puromycin selection, cells were seeded at 2×10^5/ml for 96 h in either the regular media or the media supplemented with 1mM Asp. The cell culture media was refreshed at 48h post seeding. Mean +/− SD values were from n=3 experiments, with P-values from unpaired Student’s t-test. k. Percentage of live cell number of GM12878 LCL treated with DMSO or 1μM AD, or 1μM AD+ indicated amount of sodium pyruvate for 48h. Data represent mean ± SD from three experiments, with P-values from 2way ANOVA by Tukey’s multiple comparisons test. l. Bar chart showing levels of glutamine and TCA cycle metabolites as indicated. Data represent mean ± SD from three experiments. m. Schematic of U-13C glutamine tracing. EBV infected primary B-cells were collected at 4DPI, treated with DMSO or 2μM AD for 16h, then U-13C glutamine (2mM) was applied to the cells for additional 8h. Malate(m+3) was used to measure reductive carboxylation, while Malate(m+4) was used to measure glutamine oxidation. n. Stacked bar charts of 13C labeled malate or aspartate. Data represent mean ± SD from three experiments. o. Fraction of m+4 or m+3 malate or Asp in DMSO or AD treated 4DPI EBV infected cells. Data represent mean ± SD from three experiments. P values were calculated using one-way ANOVA with Tukey’s multiple comparisons test. p. Absolute live cell number of Cas9+ GM12878 LCL cultured in 2mM glutamine or 0.2 mM glutamine, treated with DMSO or 1μM AD for 48h. Data represent mean ± SD from three experiments, with P-values from unpaired Student’s t-test.

Metabolomic analysis of intracellular amino acids in AD-treated, newly infected B-cells revealed a marked decrease in the levels of aspartate and ornithine (Fig. 6a, **Extended Data** Fig. 4). Aspartate plays a critical role in the growth and proliferation of cancer cells, serving not only as an amino acid for protein synthesis but also as a key precursor for the de novo synthesis of purines and pyrimidines^41, 42^. During the hyperproliferative phase of EBV B-cell transformation, typically occurring between 3 to 7 DPI, the infected B-cells divide approximately every 8 hours. This rapid division places a high demand on the synthesis of purines, pyrimidines, and proteins. Given that aspartate biosynthesis is closely regulated by the mitochondrial electron transport chain (ETC)^43, 44^, this decrease in aspartate could be a consequence of AD’s inhibition of oxidative phosphorylation (Ox-Phos), as shown in **in Extended Data** Fig. 5a-d..

While human plasma has low aspartate levels, certain cancer cells can uptake aspartate from their surroundings through the expression of aspartate membrane transporters^41, 45^. SLC1A3, the primary transporter of aspartate^41^, exhibits minimal to negligible transcription levels during EBV transformation in primary human B cells, particularly when compared to more common amino acid transporters like SLC1A5 (**Extended Data** Fig. 6a)^26^. Notably, SLC1A3 expression was shut down once the newly infected B-cells fully transformed into LCL (**Extended Data** Fig. 6b, **28DPI**). This implicates that de novo synthesis of aspartate might be indispensable in sustaining rapid cellular proliferation.

Aspartate biosynthesis involves the enzymes glutamate oxaloacetate transaminase 1 (GOT1) and 2 (GOT2), which function in the cytosol and mitochondria, respectively. EBV infection highly regulates B-cell GOT1 and GOT2 (**Extended Data** Fig. 6a–b)^12, 26^. In cells competent in mitochondrial respiration, aspartate synthesis primarily occurs in the mitochondria, where GOT2 catalyzes its conversion from glutamate and oxaloacetate (Fig. 6b). We therefore investigated aspartate biosynthesis in EBV B-cell transformation. We infected primary B cells with EBV for 2 hours to ensure equal viral entry. Cells were then labeled with CFSE and treated with DMSO or Aminooxyacetic acid (AOA), a dual inhibitor of GOT1 and GOT2. EBV-driven cell proliferation was assessed by CFSE intensity at 5DPI using flow cytometry. Our findings indicate that pharmaceutical inhibition of aspartate synthesis halted the B-cells proliferation induced by EBV (Fig. 6b). To further dissect the roles of GOT1 and GOT2 in EBV-transformed B-cells, we next employed CRISPR/Cas9 to knock out GOT1 or GOT2 individually in GM12878 LCL. Expressing two independent GOT1 sgRNAs consistently led to about a 30% reduction in GM12878 LCL viability. Interestingly, AD treatment synergized with GOT1 deficiency, which led to over 70% reduction in LCL viability within 48h post-treatment (Fig. 6c–d). By comparison, we found that GM12878 LCL survival is highly dependent on GOT2. Successful GOT2 KO by expressing GOT2 sgRNA #2 and #3 completely diminished GM12878 growth in comparison to cells expressing control sgRNA or GOT2 sgRNA #1, which failed to KO GOT2 (Fig. 6e–f). These data suggest that EBV transformation and LCL survival are highly dependent on functional mitochondrial aspartate biosynthesis. Importantly, we further found GOT2 but not GOT1 protein was markedly reduced in PTPMT1 KO GM12878 LCL (Fig. 6g). Our data suggest inhibiting CL biosynthesis may lead to GOT2 deficiency in EBV + LCLs.

We further tested whether adding exogenous aspartate could rescue AD-induced cell death in GM12878 LCLs. Since EBV + LCLs only express minimal level of SLC1A3, we first created a stable GM12878 LCL expressing SLC1A3 (Fig. 6h). A control cell line expressing GFP was also established. Those cells were treated with DMSO or AD. 1 mM of aspartate was used to rescue the AD effects on cell growth defects. We found exogenous aspartate was barely able to rescue the cells in the GFP group by 48 hours post-AD treatment. However, this rescue effect was enhanced in the SLC1A3-expressing cells (Fig. 6i). Similarly, exogenous aspartate was able to partially rescue the growth defects induced by PTPMT1 KO in LCLs (Fig. 6j). These data suggest that CL inhibition induced growth defects are associated with aspartate deficiency.

Two alternative cytosolic pathways related to pyruvate can compensate for aspartate biosynthesis when mitochondrial respiration is disrupted: (1) MDH1 converts malate to oxaloacetate using NAD + as an electron acceptor, with GOT1 catalyzing reverse transamination to produce aspartate. Pyruvate may enhance this pathway by converting NADH to NAD + via lactate dehydrogenase (LDH). (2) Pyruvate carboxylase (PC) can convert pyruvate to oxaloacetate. Increased LDHA and PC expressions have been noted in EBV transformation, as shown by RNAseq data (**Extended Data** Fig. 6)^26^. We therefore examined if exogenous pyruvate could restore AD-induced aspartate deficiency. Yet, adding exogenous pyruvate did not prevent cell death in GM12878 LCLs with AD (Fig. 6k), indicating neither PC nor MDH1 pathways could compensate for aspartate biosynthesis in EBV + LCLs under CL inhibition.

We further investigated the carbon source supporting aspartate biosynthesis during EBV B-cell transformation. Our metabolomic analysis of newly infected B-cells revealed that AD treatment generally increased TCA cycle metabolite levels, contrary to PierA treatment, which shut down the upper TCA cycle (Fig. 6l). Notably, a significant increase in glutamine and citrate was observed in AD-treated cells, suggesting the possibility that citrate might produce oxaloacetate via ATP-citrate lyase (ACLY) in the cytosol. Given that citrate is primarily generated through glutaminolysis and reductive carboxylation in cells with impaired respiration, glutamine utilization might also contribute to aspartate maintenance in AD-treated newly infected B-cells. To explore this hypothesis, we conducted an isotopic tracing study using glutamine labeled with uniformly distributed 13-carbon atoms (U-13C glutamine). We collected newly transformed B-cells at 4DPI and subsequently treated them with either DMSO or AD for 24 hours. Then, we introduced U-13C glutamine to these cells for an additional 8-hour period to facilitate 13C integration. In this experiment, the oxidation of glutamine results in the generation of M + 4 labeled compounds, whereas its reductive carboxylation leads to the formation of M + 3 labeled compounds (Fig. 6m). Notably, after 8 hours, about 82% of the malate and 69% of the aspartate derived from glutamine were labeled in DMSO-treated cells (Fig. 6m), suggesting that glutamine is the major carbon source supporting aspartate biosynthesis in newly infected B-cells. With AD treatment, there was not only an increase in malate abundance but also in the proportion of glutamine-derived malate, reaching about 85% (Fig. 6n). Treatment with AD led to a 20% increase in the production of malate from glutamine oxidation (M + 4) and a concurrent decrease in M + 3 malate produced via glutamine’s reductive carboxylation (Fig. 6n–o). Although there was a significant overall reduction in aspartate derived from glutamine, the proportion of M + 4 aspartate showed a modest increase (Fig. 6n–o). The overall M + 4 aspartate was much higher than M + 3 aspartate (Fig. 6o). Aligned with the isotopic tracing data, glutamine restriction sensitized GM12878 cells to AD treatment (Fig. 6p). Collectively, these data indicate that glutamine serves as a primary carbon source for aspartate biosynthesis and CL inhibition destabilizes GOT2 which results in aspartate deficiency in EBV-transformed B-cells.

## Discussion

Shortly after entry, EBV actively initiates aerobic glycolysis in B cells through the translational activation of all glycolytic enzymes and quickly downregulates TXNIP, a strong GLUT1 negative regulator. Glucose consumption and lactate release are evident at 2 DPI and peak at 4 DPI^12^. EBV early infection also boosts key fatty acid synthesis enzymes, ACACA and FASN, to convert acetyl-CoA derived from glycolysis into palmitate, facilitating lipogenesis^13^. Here, we show that EBV concurrently activates CL biosynthesis within 4 days post-infection. We speculate that the initial increase in flux of glycerol-3-phosphate from glycolysis and acyl groups from lipogenesis may boost CL biosynthesis resulting in mitochondrial remodeling. Previous research has shown significant growth defects in newly infected EBV cells when glucose in the culture medium is replaced with galactose. How this substitution affects dynamic mitochondrial CL biosynthesis remains to be further addressed.

MYC is a master regulator of metabolism^46^. EBNA2 and MYC jointly activate glycolysis, Ox-Phos, 1C metabolism, and lipogenesis during EBV B-cell transformation^28^. Despite ChIP-seq analysis in LCLs revealing MYC co-occupancy at the promoters of CRLS1, our data suggest that EBNA2 may independently initiate CRLS1 expression in LCLs. We speculate that EBNA2 plays a dominant role in CRLS1 expression, with this effect potentially being further augmented by MYC. Additionally, it’s noteworthy that EBNA2-driven PTPMT1 activation begins at 2 DPI and increases significantly after 7 DPI in newly infected cells. This aligns with the onset of LMP1, which mimics CD40 and activates key signaling pathways including NF-κB, MAPK, and PI3K/AKT^28, 47, 48^. LMP1 notably upregulates GLUT1 via canonical NF-κB and PI3K/AKT pathways^49^ and enhances glycolysis by inducing HIF1α through MAPK signaling^19^. We speculate that PTPMT1 could be a critical LMP1 target, whose activation could better utilize LMP1-induced glycolysis to facilitate mitochondrial remodeling and finalize the transformation.

CL contributes to the structural integrity and functionality of mitochondrial cristae. While there is ongoing debate regarding CL’s cone-shaped structure’s direct contribution to cristae curvature, it interacts with numerous mitochondrial protein complexes to support cristae formation. The Mitochondrial Contact Site and Cristae Organizing System (MICOS) play crucial roles in shaping and maintaining cristae structure^50^. Notably, EBV transcriptionally upregulates MICOS genes, including MIC60, during B-cell transformation^26^. The loss of CL is associated with MICOS disruption, leading to cristae structure loss and mitochondrial dysfunction, as observed in fibroblasts from Barth Syndrome patients^50^. How EBV-driven CL biosynthesis coordinates with MICOS to support cristae biogenesis remains to be investigated. Furthermore, the dimerization of F1Fo-ATP synthase and the integration of complex II into the respirasome are crucial for cristae curvature formation. CL is known to bind to and stabilize all ETC complexes and ATP synthase (Complex V), modulating their stability and function^51–55^. Our findings indicate that PTPMT1 deficiency results in a highly disordered tubular structure in the mitochondria of newly infected B-cells. We are tempted to speculate that disruptions in the assembly and integration of protein complexes into the cristae may contribute to the observed disorder. Supporting this hypothesis, our BN-PAGE data revealed a deficiency in Complex II, where the assembly of the SDHA subunit was disrupted due to PTPMT1 deficiency in EBV-transformed B-cells.

We discovered that blocking CL biosynthesis leads to the destabilization of mitochondrial 1C enzymes targeted by EBV, resulting in a significant reduction of NADPH production. This finding reveals a previously unrecognized role of CL in maintaining mitochondrial 1C metabolism. As mentioned above, CL’s ability to bind to and interact with a wide variety of mitochondrial protein complexes was well documented. The interaction between CL and proteins involves strong hydrophilic interactions facilitating the binding between its negative charged glycerol head group and various amino acid residues of the protein^23^. We speculate that during EBV B-cell transformation, CL in the IMM probably binds to mitochondrial 1C enzymes, which may promote 1C enzymes to form metabolons to meet the increased demand for nucleotides and NADPH. Therefore, further biochemical and functional studies are essential to thoroughly understand the interactions between CL and mitochondrial 1C enzymes and to elucidate how these interactions contribute to EBV-induced lymphomagenesis.

Our research has, for the first time, demonstrated that aspartate, crucial for protein and nucleotide synthesis during EBV-driven cell proliferation, is predominantly synthesized in mitochondria from glutamate and oxaloacetate by GOT2. This pathway represents a significant metabolic vulnerability in EBV-transformed B-cells. Notably, besides maintaining respiration, CL plays a vital role in stabilizing GOT2 throughout the transformation process, thereby ensuring sustainable aspartate synthesis. Interestingly, while some of the most aggressive cancers, characterized by compromised respiration, depend on GOT1-driven pathways to compensate for aspartate synthesis, our findings from pyruvate rescue experiments and isotopic tracing suggest that EBV-transformed B-cells lack the ability to utilize these major alternative pathways. This implies that directly targeting GOT2 could be considered as an effective strategy for treating lymphomas associated with EBV B-cell transformation. Furthermore, our data reveals that in the initial stages of B-cell transformation, glutamine oxidation via the TCA cycle, is the primary source of aspartate synthesis. The combination of glutamine restriction and AD treatment displayed synthetic lethality, underscoring a potential therapeutic vulnerability. These insights suggest that co-targeting CL biosynthesis and glutaminolysis may offer innovative approaches to induce aspartate depletion, presenting a promising avenue for the treatment of EBV-associated lymphomas.

AD, a bis-biguanide compound commonly utilized as an oral antibiotic and for preventing gingivitis^56^, has been found to be particularly effective against the EBV + nasopharyngeal carcinoma cell line C666–1, but not in non-transformed cell types such as GM05757, HNEpC, or NIH/3T3^57^. Our findings underscore that AD significantly suppresses EBV B-cell transformation. Importantly, it exhibits a pronounced synthetic lethal effect when used in conjunction with SHIN1, a SHMT inhibitor in EBV-transformed B-cells and related B-cell lymphomas. Given the rapid clearance of SHIN1 in vivo, it would be of interest to test SHMT antagonist SHIN2, a second generation SHIN1 derivative with improved in vivo pharmacokinetic properties in EBV lymphoma mouse xenografts models^14, 58^.

In conclusion, our findings reveal a key role of CL biosynthesis in generating EBV-specialized mitochondria to meet the energetic, biosynthetic, and redox demands during B-cell lymphomagenesis. Targeting CL biosynthesis and employing CL-related synergistic combinations could serve as a promising new therapeutic approach for treating EBV-associated cancers.

## Methods

### Cell lines and reagents

EBV + P3HR-1 and Daudi BL cell lines were obtained from ATCC. EBV + Mutu I BL was obtained from Dr. Ben Gewurz (originally a gift from Jeff Sample). EBV + Rael BL was obtained from Dr. Lisa Guilino Roth. EBV + GM12878, GM12892, GM12881, GM14890, and GM15892 LCLs were obtained from the Coriell Institute. 2-3-3 E2HT LCLs, expressing EBNA2 fused to an estrogen receptor 4-hydroxytamoxifen (4HT)-binding domain, were obtained from Dr. Bo Zhao. In the presence of 1 μM 4HT, EBNA2HT localizes to the nucleus; without 4HT, it relocalizes to the cytosol and becomes destabilized. To remove 4HT, cells underwent five washes with 4HT-free media, the last two washes lasting 30 minutes each before re-seeding at 300,000 cells per mL. Cells were then grown for an additional 48 hours before cell lysate preparation. The conditional P493–6 LCLs, a gift from Dr. Ben Gewurz, contain a conditional EBNA2-HT allele and an exogenous Tet-OFF MYC allele. Cells were washed three times with PBS, then seeded at 0.3 million/mL in RPMI-1640 media with 10% doxycycline-free FBS, and treated under various conditions for 48h as specified. To culture P493–6 cells at a low MYC state, they were grown without 4-hydroxytamoxifen (4HT, SML1666, Sigma-Aldrich) and with 1 mM doxycycline (HY-N0565, MedChemExpress). For a high MYC BL-like state, both doxycycline and 4HT were removed. For a high EBNA2 LCL state, cells were treated with 1 μg/mL doxycycline and 1 μM 4HT. For a high MYC and EBNA2 state, cells received 1 μM 4HT but no doxycycline. HEK-293 T cell line was obtained from ATCC. All the BL, DLBCL, LCL, and PEL cell lines, as well as primary B cells, were cultured in RPMI 1640 medium (Gibco, Life Technologies) with 10% fetal bovine serum (FBS, Gibco) or with dialyzed FBS where indicated (Gibco). HEK-293 T cells were cultured in Dulbecco’s Modified Eagle’s Medium (DMEM, Gibco) with 10% FBS. To obtain the stable Streptococcus pyogenes Cas9 expression, cell lines were treated with lentiviral transduction and blasticidin (5 μg/mL, ant-bi-1, InvivoGen) selection. To select the transduced cells, puromycin (3 μg/mL, A11138–03, Gibco) or hygromycin (100 μg/mL, 10687010, ThermoFisher) were added to the post-infection cells. All Cells were cultured at 37° in 5% CO2 incubator.

Cells were treated with 1 μM alexidine dihydrochloride (AD, A4542, ApexBio) at a density of 2×10^5/mL for 48 hours, refreshing AD after 24 hours and diluting the cells 1:1 with culture media. AD treatment details are specified in figure legends. For antioxidant rescue analysis with N-acetyl-L-cysteine (NAC, 7874, Tocris) and L-glutathione reduced (GSH, 70-18-8, Millipore), GM12878 cells were co-treated with 1 μM AD and either 1.5 mM NAC or 2 mM GSH, refreshing treatments after 24 hours. GOT1 or G6PD knockout GM12878 LCLs, after puromycin selection, were treated with DMSO or 1μM AD for 48 hours. Similarly, GM12878 LCLs expressing GFP or SLC1A3, post-puromycin selection, received DMSO, 1μM AD, or 1μM AD plus 1mM L-aspartate (A1330000, Sigma-Aldrich) for 48 hours. For pyruvate supplementation studies, GM12878 LCL cells were treated with DMSO or 1μM AD and 0, 1, or 5 mM sodium pyruvate (P5280, Sigma-Aldrich) for 48 hours.

Live cell counts following AD treatment with or without 10μM SHIN1 (S6392, Selleckchem) were performed on GM12878 LCL, GM15892 LCL, Mutu I BL, or Daudi BL, using DMSO, 1μM AD, 10μM SHIN1, or a combination of 1μM AD and 10μM SHIN1 for 48 hours. For studies on the effect of glutamine restriction with AD treatment, GM12878 LCLs were washed with PBS, resuspended in glutamine-free RPMI-1640 (21870–076, Gibco) with 10% dialyzed FBS, seeded at 2×10^5/mL, supplemented with 2 mM or 0.2 mM L-Glutamine (25030–149, Gibco), and treated with DMSO or 1μM AD for 48 hours.

### Human Primary B cells isolation

Discarded, de-identified leukocyte fractions left over from platelet donations were obtained from the Brigham and Women’s Hospital Blood Bank. Blood cells were collected from platelet donors following institutional guidelines. Since these were de-identified samples, the gender was unknown. Our studies on primary human blood cells were approved by the Tufts University Institutional Review Board (Tufts IRB: STUDY00004385). Primary human B cells were isolated by negative selection using RosetteSep Human B Cell Enrichment and EasySep Human B cell enrichment kits (Stem Cell Technologies), according to the manufacturers’ protocols. B cell purity was confirmed by plasma membrane CD19 positivity through FACS. Cells were then cultured with RPMI 1640 with 10% FBS.

### EBV production and concentration

The EBV B95–8 strain was generated from B95–8 cells engineered for inducible ZTA expression (a gift from Dr. Ben Gewurz). The activation of EBV lytic cycle was achieved by treating the cells with 1 μM of 4HT for 24 hours. Subsequently, the 4HT was removed, and the cells were cultured in RPMI medium supplemented with 10% FBS, devoid of 4HT, for an additional 96 hours. The viral supernatants obtained were then cleared of producer cells by passing through a 0.45 μm filter. The viral titer was assessed using a transformation assay. Similarly, the P3HR-1 strain of EBV was obtained from a P3HR-1 cell line that expresses 4HT-inducible ZTA-HT and RTA-HT alleles, generously provided by Dr. Ben Gewurz. The induction process involved treating P3HR1 ZHT/RHT cells with 1 μM of 4HT for a 24-hour period. Afterwards, the culture medium was replaced with fresh RPMI/FBS medium, and the cultures were allowed to incubate for 96 hours to collect the virus-rich supernatants. These supernatants were then filtered using a 0.45 μM filter for purification. The supernatant was transferred to an ultracentrifuge tube (326823, Beckman Coulter) and centrifuged at 25,000 rpm for 2 h at 4°C in an ultracentrifuge (OPTIMA XPN-100, Beckman Coulter). The viral pellet was resuspended and aliquoted in PBS with 2% dialyzed FBS, stored at − 80°C until infection. The genomic DNA of virus was quantified by PCR targeting the BALF5 gene from the extracted viral genome. This quantification was used to standardize the virus amounts for cell infection experiments.

### Transmission electron microscopy (TEM)

A mixture of 2.5% paraformaldehyde, 5% glutaraldehyde, and 0.06% picric acid in 0.2 mol/L Cacodylate buffer was freshly prepared before use, then the above mixture was diluted 1:1 with dH2O. To Fix primary B cells, 1 million cells were collected and washed with Dulbecco’s Phosphate Buffered Saline (DPBS, 14190, Gibco) one time, remove the residue buffer, then gently add the diluted fixative to the primary B cells. The cells were fixed at RT for 1h. The cells were then post-fixed for 30 min in 1% Osmium tetroxide (OsO4)/1.5% potassium ferrocyanide (KFeCN6), washed in water 3x and incubated in 1% aqueous uranyl acetate for 30 minutes. Samples were then washed twice in water and dehydrated in grades of alcohol (5min each; 50%, 70%, 95%, 2× 100%). Cells were removed from the dish in propyleneoxide, pelleted at 3000 rpm for 3 minutes and infiltrated for 2 hours in a 1:1 mixture of propyleneoxide and TAAB Epon (Marivac Canada Inc. St. Laurent, Canada). Samples were subsequently embedded in TAAB Epon and polymerized at 60 degrees C for 48 hrs. Ultrathin sections (about 60nm) were cut on a Reichert Ultracut-S microtome, picked up on to copper grids stained with lead citrate and examined in a JEOL 1200EX transmission electron microscope or a TecnaiG2 Spirit BioTWIN. Images were recorded with an AMT 2k CCD camera.

### CRISPR/Cas9 editing

CRISPR/Cas9 knock-out was performed in cells with stably Cas9 expression, using Broad Institute Brunello or Avana library sgRNA sequences as listed in Table S1. CRISPR/dCas9 interference (CRISPRi) was performed in GM12878 LCL expressing dCas9 fused with KRAB. Single guide RNAs against targeting upstream CRLS1 enhancers were designed using GPP sgRNA Designer at the Broad Institute. sgRNA oligos were obtained from Integrated DNA Technologies and cloned into the pLentiGuide-Puro vector (Addgene plasmid #52963, a gift from Feng Zhang). Lentiviruses were produced in HEK-293 cells by co-transfection of pLentiGuid-puro with psPAX2 and VSV-G packaging. At 24 hours post transfection, cell culture media was changed to RPMI-1640 + 10% FBS. Two rounds of lentiviral transduction were performed at 48 and 72 hours post plasmids transfection. Cells were selected by puromycin (3 μg/ml), added 48 hours post-transduction. Depletion of target gene encoded protein expression was confirmed by immunoblot.

### cDNA rescue

The PTPMT1 cDNA with silent PAM site mutations was purchased from IDT and was inserted into pLX-TRC313 (a gift from John Doench) by HifiAssembly (New England Bioloabs). GM12878 −Cas9 with stable C-terminal V5 epitope-tagged PTPMT1 cDNA expression was established by lentiviral transduction and hygromycin selection as described above. Ten days post hygromycin selection, PTPMT1-V5 expression was confirmed by immunoblot. The sequence of PTPMT1 rescue cDNA is listed below. sg PTPMT1 targeting sequences are highlighted in underlined bold. PAM sequences are underlined. Mutation sites are indicated in italics bold. Overlapping sequences for HifiAssembly reaction are highlighted in underlined.

>PTPMT1 cDNA

TCTTCCATTTCAGGTGTCGTGAGGCTAGCATGGCGGCCACCGCGCTGCTGGAGGCCGGCCTGGCGCGGGTGCTCTTCTACCCGACGCTGCTCTACACCCTGTTCCGCGGGAAGGTGCCGGGTCGGGCGCACCGGGACTGGTACCACCGCATCGACCCCACCGTGCTGCTGGGCGCGCTGCCGTTGCGGAGCTTGACGCGCCAGCTGGTACAGGACGAGAACGTGCGCGGGGTGATCACCATGAACGA**A**GAGTACGAGACGAGGTTCCTGTGCAACTCTTCACAGGAGTGGAAGAGACTAGGAGTCGAGCAGCTGCGGCTCAGCACAGTAGACATGACTGGGATCCCCACCTTGGACAACCTCCAGAAGGGAGTCCAATTTGCTCTCAAGTACCAGTCGCTGGGCCAGTGTGTTTACGTGCATTGTAAGGCTGGGCGCTCCAGGAGTGCCACTATGGTGGCAGCATACCTGATTCAGGTGCACAAATGGAGTCCAGAGGAGGCTGTAAGAGCCATCGCCAAGATCCGGTCATACATCCACATCAGGCCTGGCCAGCTGGATGTTCTTAAAGAGTTCCACAAGCAGATTACTGCACGGGCAACAAAGGATGGGACTTTTGTCATTTCAAAGACAGATATCGGTAAGCCTATCCCTAACCCTC

### Mitochondria isolation

The mitochondria were isolated following the manufacturer’s protocols of Mitochondria Isolation Kit for Cultured Cells (89874, Thermo Fisher Scientific). Specifically, 20 million cells were harvested into a 2.0 mL microcentrifuge tube, centrifuged at 850 g for 2 minutes, the supernatant was removed and then the cell pellet was added with 800 μL of Mitochondria Isolation Reagent A containing EDTA-free protease inhibitor (cOmplete^™^, Millipore Sigma). Gently vortex and incubate on ice for exactly 2 minutes. 10 μL of Mitochondria Isolation Reagent B was then added to the mixture and incubated on ice, fully vortexing the mixture at every minute. After 5 minutes, 800 μL of Mitochondria Isolation Reagent C containing EDTA-free protease inhibitor was included, gently inverted tube several times before centrifuging at 700 g for 10 minutes at 4°C. Transfer the supernatant to a new, 2.0 mL tube and centrifuge at 12,000 g for 15 minutes at 4°C. The obtained mitochondrial pellet was resuspended in 100 μL of Mitochondria Isolation Reagent C containing EDTA-free protease inhibitor. The protein content of mitochondria was further measured by Qubit 4 Fluorometer (Q33226, Thermo Fisher Scientific) with Qubit Protein Assay kit (Q33212, Thermo Fisher Scientific).

### Blue-Native (BN) gel electrophoresis and immunodetection.

#### Sample preparation.

The BN gel electrophoresis and Immunoblot was conducted as previously described^59^. The NativePAGE sample prep kit (BN2008, Thermo Fisher Scientific) was used to make the mitochondrial Sample buffer cocktail. 50 μg mitochondrial protein was mixed with 5 μL 4× NativePAGE sample buffer, 8 μL 5% digitonin, and 7 μL water in the kit. Then we incubated the solubilized mitochondria on ice for 20 min. After that, solubilized mitochondria were then centrifuged at 20,000 g for 10 min at 4°C. 15 μL supernatant was transferred into a new tube and fully mixed with 2 μL Coomassie G-250 sample additive in kit.

#### Electrophoresis.

Each well (NativePAGE 3–12% gradient gel, BN2011, BX10, Thermo Fisher Scientific) was gently washed with 1 mL of dark blue 1X cathode buffer (BN2002, Thermo Fisher Scientific). Subsequently, 15 μL of mitochondrial sample was loaded into the gel. The inner chamber was filled with 1X dark blue cathode buffer, and 600 mL of 1X running buffer (BN2002, Thermo Fisher Scientific) was added to the outer chamber. Electrophoresis took place in an XCell SureLock Electrophoresis-Cell (EI0001, Novex) at a constant voltage of 150 V for 30 minutes, with the current limited to 15 mA. Following this, the buffer in the inner chamber was replaced with light blue buffer (created by mixing 20 mL of dark blue 1X cathode buffer with 180 mL of distilled water). Electrophoresis continued at 250 V for 60 minutes.

#### Immunoblot.

The BN-PAGE gel was gently washed with water for 5 minutes to remove the cathode buffer. It was then placed in bicarbonate transfer buffer (10 mM NaHCO_3_, 3 mM Na_2_CO_3_) for a 15-minute incubation. The PVDF membrane was activated by immersion in 100% methanol for 2 seconds and then washed with water for 5 minutes, followed by a 5-minute incubation in bicarbonate transfer buffer. The transfer was carried out at a constant current of 300 mA for 1 hour in the cold room. Following the transfer, the membrane was rinsed with PBS and then fixed with 8% acetic acid for 5 minutes. The membrane was subsequently washed with water three times, each for 5 minutes. To remove the Coomassie blue, the membrane underwent shaking with methanol three times, each for 5 minutes. This was followed by a water wash, also three times for 5 minutes each. The membrane was incubated with 5% milk in PBS-Tween 20 (PBST) for blocking for 1 hour. The membrane was washed with PBST three times, each for 5 minutes, and then incubated with primary antibodies for the electron transport chain Complexes I, II, III, IV, and Ox-Phos overnight at 4°C. The next day, the membrane was washed with PBS three times, each for 5 minutes, followed by a 1-hour incubation with the secondary antibody and then washed three times with PBST for 5 minutes each. Chemiluminescent detection was performed on the membranes by LI-COR XF system.

### Lipidomic profiling analysis

The intracellular lipidomic profiling was performed as described^60^. Newly isolated human B-cells were mock infected or infected with EBV at a MOI of 1 for 5 days. B-Cells were counted and pelleted at 1,200 rpm for 5 minutes at 4°C with an equal number of cells in each sample. Lipidomic profiling was performed as described previously^60, 61^. They were then resuspended in 200 μL of HPLC-grade water (270733, Sigma-Aldrich) and mixed vigorously with 2.5 mL of HPLC-grade methanol (A456, Fisher Scientific) in glass tubes. Following this, 5 mL of methyl tert-butyl ether (MTBE, 1634-04-4, Supelco) was added, and the samples were agitated for 1 hour at room temperature. To separate phases, 1.5 mL of water was added, and after vigorous vortexing, the samples were centrifuged at 1000 × g for 10 minutes at room temperature. The upper phase was then dried a speed vacuum concentrator (Savant SPD 1010, Thermofisher Scientific) for 4h at RT and stored at − 80°C.

For analysis, samples were reconstituted in 35 μL of a 1:1 mixture of LCMS-grade isopropanol and methanol, and subjected to liquid chromatography-mass spectrometry (LC-MS) as previously outlined, employing a high-resolution hybrid QExactive HF Orbitrap mass spectrometer (Thermo Fisher Scientific) set to data-dependent acquisition mode (Top 8) with the capability of switching between positive and negative ion polarities. Lipid species identification and quantification were performed using the LipidSearch 4.1.30 software (Thermo Fisher Scientific), leveraging an internal database comprising ≥ 20 major lipid classes and ≥ 80 subclasses. For verifying signal linearity, a pooled sample was created by combining 5 μL from each sample, which was then diluted with a 1:1 mixture of isopropanol and methanol to generate dilutions of 0.3x and 0.1x, alongside a blank. These dilutions underwent analysis, and for each lipid species within this series, the Pearson correlation coefficient between ion count and sample concentration was computed. Only lipids exhibiting a correlation coefficient (r) greater than 0.9 were retained for final analysis. The abundance of individual lipid species was normalized against the total ion count of the sample. Using R, lipids were categorized by class, and the total ion intensity for each lipid class in each sample was calculated.

### Intracellular metabolite profiling

The intracellular metabolites profiling was performed as described^62^. Newly infected B-cells collected at 4DPI were washed 3 times with PBS and counted. 6 million cells were seeded into a T25 flask with 20 mL RPMI-1640 with 10% dialyzed FBS. Cells were incubated with DMSO, 2uM AD, or 100nM ofPiericidin A (MedChemExpress, 2738-64-9) for 24h. The cells were counted and washed 3 times with pre-chilled PBS. The cell pellet was fully resuspended with 100uL PBS by vortex, the metabolism was quenched by adding 3.3 mL of dry ice-cold 80% aqueous methanol (A456, Fisher Scientific), and kept at −80°C overnight. The lysate was centrifuged at 21,000 g for 15 min at 4°C. The supernatants were obtained and dried by a speed vacuum concentrator (Savant SPD 1010, Thermofisher Scientific) for 4h at RT. Samples were re-suspended using 20 uL HPLC grade water for mass spectrometry. 5–7 μL were injected and analyzed using a hybrid 6500 QTRAP triple quadrupole mass spectrometer (AB/SCIEX) coupled to a Prominence UFLC HPLC system (Shimadzu) via selected reaction monitoring (SRM) of a total of 300 endogenous water soluble metabolites for steady-state analyses of samples and 150 endogenous metabolites for 13C/15N isotopomer flux tracing. Some metabolites were targeted in both positive and negative ion mode for a total of 311 SRM transitions using positive/negative ion polarity switching. ESI voltage was + 4950V in positive ion mode and − 4500V in negative ion mode. The dwell time was 3 ms per SRM transition and the total cycle time was 1.55 seconds. Approximately 9–12 data points were acquired per detected metabolite. Samples were delivered to the mass spectrometer via hydrophilic interaction chromatography (HILIC) using a 4.6 mm i.d × 10 cm Amide XBridge column (Waters) at 400 μL/min. Gradients were run starting from 85% buffer B (HPLC grade acetonitrile) to 42% B from 0–5 minutes; 42% B to 0% B from 5–16minutes; 0% B was held from 16–24 minutes; 0% B to 85% B from 24–25 minutes; 85% B was held for 7 minutes to re-equilibrate the column. Buffer A was comprised of 20 mM ammonium hydroxide/20 mM ammonium acetate (pH = 9.0) in 95:5 water:acetonitrile. Peak areas from the total ion current for each metabolite SRM transition were integrated using MultiQuant v3.0.2 software (AB/SCIEX). Metabolites with p-values < 0.05, log2(fold change) > 1 or <−1 were used for pathway analysis using MetaboAnalyst 5.0 (https://www.metaboanalyst.ca/MetaboAnalyst/ModuleView.xhtml).

### U-13C-Glutamine tracing

EBV infected primary B-cells were collected at 4DPI, treated with DMSO or 2μM AD for 16h, then U-13C glutamine was applied to the cells. Ten million cells were cultured in glutamine-free media containing 10% dialyzed FBS- and 2 mM ^13^C5-L-Glutamine (184161-19-1, Cambridge Isotope Laboratories) for 8 h. Cell samples were collected and processed as mentioned in intracellular metabolite profiling. Metabolic flux analysis was performed as described^63^.

### Seahorse mitochondrial stress test

The Agilent Seahorse XF Assay was conducted as described preiously^12^. Specifically, the sensor cartridge was first hydrated with water overnight and incubated with XF Calibrant for 1h. Add 12 μL Cell-Tak solution (1.3 mL of 0.1M sodium bicarbonate, 11.2 μL of 0.1M NaOH, 22.4 μL of Cell-Tak solution) to each well of the V7-PS 96-well cell culture plate. The Cell-Tak solution was washed with sterile water twice and 0.25 million primary B cells (resuspension in 180 μL of RPMI-1640 with 10% FBS and 5 mM pyruvate) were seed on a Seahorse plate. Then the cells were placed in a non-CO2 37°C for 30 minutes. The Oxygen consumption rates (OCR) were simultaneously recorded by a Seahorse XFe96 Analyzer (Agilent). The cells were sequentially probed by 20 μL of 3.5 μM oligomycin, 20 μL of 2 μM CCCP, and 20 μL of 100 nM piericidin A. For Seahorse in GM12878 cells, cells were seeded as 0.1 million/well in a 96-well cell culture plate. Data were analyzed by Seahorse Wave Desktop Software (Agilent).

### Flow cytometry analysis

The mitochondrial mass was determined by the MitoTracker Green FM (M7514, Thermo Fisher Scientific) and the mitochondrial membrane potential was determined by the fluorescence intensity of TMRM (Tetramethyl-rhodamine methyl ester perchlorate, T668, ThermoFisher Scientific) following the manual. 1×10^6^ of Cells were collected and resuspended in 500 μL cell culture media with 1.5 μL 100 μM of MitoTracker Green or TMRM. Cells were then incubated in 37°C incubator for 30 min. Then cells were washed once with 1×PBS and resuspended in PBS buffer with 2% FBS for FACS. For cell viability analysis, cells were washed and resuspended with PBS buffer with 2% FBS. Then cells were incubated with 1 μM of 17-Aminoactinomycin D (7AAD, A1310, Invitrogen) for 5 min before analyzing. For CFSE (C345544, Invitrogen) cell proliferation staining, 10 millions of primary B cells were resuspended in PBS with 0.1% BSA, then the cells were mixed with the same volume of 1μM CFSE for 10 min at 37°C. Cells were then neutralized by prechilled 10% FBS RMPI-1640 for 5 min. After washing the cell with culture media, cells were resuspended and infected with virus. 1h after infection, cells were treated with 100 μM Aminooxyacetic acid (AOA), CFSE were analyzed at 5DPI. For cell cycle analysis, cells were fixed with ice-cold 70% ethanol for at least 24h. At the day for analysis, cells were centrifuged at 3000 rpm for 10 min to remove ethanol, and resuspended in PBS buffer. Cells were incubated in 1 mL of staining buffer (propidium iodide, 5 μg/ml; RNase A, 40 μg/ml; 0.1% Triton X-100 in PBS) at room temperature for 30 min in dark. For MitoSOX analysis, 1 million cells were collected and washed with HBSS (Hank’s Balanced Salt Solution, Gibco) buffer, then incubated with 1uM MitoSOX^™^ Green (M36006, Thermo Fisher Scientific) for 30 min. cells gently washed with warm HBSS buffer. Flow cytometry was performed on a BD FACS Calibur instrument. Data were analyzed with FlowJo V10.

### Quantitative real-time PCR (qRT PCR)

Extraction of total RNA from cells was conducted by the RNeasy Mini Kit (Qiagen). RNase-Free DNase Set (Qiagen) were used to remove genomic DNA and SYBR Green RNA-to-CT 1-Step Kit (Applied Biosystems) were applied to assemble the qRT PCR reaction. Primer sequences are listed in the Supplementary Table. Samples were run in technical triplicates. The relative expression was calculated using the 2^−ΔΔCt^ method, and the data was normalized to internal control β-actin mRNA levels.

### DNA extraction and qPCR

The total DNA from the intracellular was extracted by the Blood & Cell culture DNA mini kit (Qiagen #13362). Extracted DNA was diluted to 10 ng/μL and quantified by qPCR for the EBV BALF5 in the Supplementary Table. For quantification of intracellular EBV genome copy number, standard curves of BALF5 were set as serial dilution of a pHAGE-BALF5 miniprep DNA at 25 ng/μL. Viral DNA copy number was calculated according to the inputting sample Ct and the standard curve as described previously^64^. For quantification of mitochondrial DNA, the total DNA samples were used for qPCR analysis of MT-ND1. The relative expression was calculated using the 2-ΔΔCt method, and the data was normalized to β-actin. The fold change was calculated by normalizing data points to uninfected control (0DPI) DNA levels.

### Western blot analysis

Immunoblot analysis was performed according to the previous instructions^14^. Cell lysates were prepared by incubating cells in 1× Laemmli buffer at 95°C for 5 min. For the target protein anchoring to the membrane like SLC1A3, and the electron transport chain, Cell lysates were incubated at 37°C for 1h. Lysate Samples were separated by SDS-PAGE electrophoresis, transferred onto the nitrocellulose membranes, blocked with 5% milk in TBST buffer for 1 h, and then probed with relevant primary antibodies at 4°C overnight. The next day, the membranes were incubated with secondary antibody for 1 h. Blots were then developed by incubation with ECL chemiluminescence (Millipore) and images were captured by Licor Fx system. Bands intensities were measured where indicated by Image Studio Lite Version 5.2. All antibodies used in this study were listed in Supplementary Table S1.

### Cell viability analysis

Absolute live cell counts were determined using the Countess 3 automatic counter with Trypan Blue (15250061, ThermoFisher Scientific) staining. For growth curve analysis, cells were seeded at a density of 2×10^5/mL in 12-well tissue culture plates. For growth curve analysis of CRISPR/Cas9 knock-out cells, cells were seeded at day 5 post-puromycin selection. The KO effects were confirmed by immunoblotting. The absolute live cell numbers were adjusted based on the culture splitting factors.

### Statistical analysis

Unless otherwise indicated, all bar graphs and line graphs represent the arithmetic mean of three independent experiments (n = 3), with error bars denoting standard deviations. Data were analyzed using two-tailed paired Student t test or analysis of variance (ANOVA) with the appropriate post-test using GraphPad Prism7 software. Metabolic pathway analysis was performed using MetaboAnalyst 3.0.

### Graphics

Figures were drawn with GraphPad, Biorender, Microsoft Powerpoint, and ggplot2 in R.

## Data Availability

The authors have stated that the data underpinning the results of this study can be found within the main text and the Supplementary Information files. Additionally, this data will be provided by the corresponding author upon receiving a reasonable request. Source data is available as a source data file. Other ChIP-seq or ChIA-PET data were obtained from GEO: GM12878 LCL POLR2A ChIA-PET, GSE72816; EBNA2 ChIP-seq, GSE29498; MYC ChIP-seq, GSM822290; MAX ChIP-seq, GSM935518; H3K4me1 ChIP-seq, GSM733772; H3K4me3, GSE95899; H3K27ac ChIP-seq, GSM733771; P300, GSM803387. RNA sequencing data were obtained from GSE125974.
